# Concrete Defect Localization Based on Multilevel Convolutional Neural Networks

**DOI:** 10.3390/ma17153685

**Published:** 2024-07-25

**Authors:** Yameng Wang, Lihua Wang, Wenjing Ye, Fengyi Zhang, Yongdong Pan, Yan Li

**Affiliations:** School of Aerospace Engineering and Applied Mechanics, Tongji University, Shanghai 200092, China; 2130875@tongji.edu.cn (Y.W.); 2210655@tongji.edu.cn (W.Y.); 2030888@tongji.edu.cn (F.Z.); ypan@tongji.edu.cn (Y.P.); liyan@tongji.edu.cn (Y.L.)

**Keywords:** convolutional neural networks, concrete structures, array ultrasonic testing, defect localization

## Abstract

Concrete structures frequently manifest diverse defects throughout their manufacturing and usage processes due to factors such as design, construction, environmental conditions and distress mechanisms. In this paper, a multilevel convolutional neural network (CNN) combined with array ultrasonic testing (AUT) is proposed for identifying the locations of hole defects in concrete structures. By refining the detection area layer by layer, AUT is used to collect ultrasonic signals containing hole defect information, and the original echo signal is input to CNN for the classification of hole locations. The advantage of the proposed method is that the corresponding defect location information can be obtained directly from the input ultrasonic signal without manual discrimination. It effectively addresses the issue of traditional methods being insufficiently accurate when dealing with complex structures or hidden defects. The analysis process is as follows. First, COMSOL-Multiphysics finite element software is utilized to simulate the AUT detection process and generate a large amount of ultrasonic echo data. Next, the extracted signal data are trained and learned using the proposed multilevel CNN approach to achieve progressive localization of internal structural defects. Afterwards, a comparative analysis is conducted between the proposed multilevel CNN method and traditional CNN approaches. The results show that the defect localization accuracy of the proposed multilevel CNN approach improved from 85.38% to 95.27% compared to traditional CNN methods. Furthermore, the computation time required for this process is reduced, indicating that the method not only achieves higher recognition precision but also operates with greater efficiency. Finally, a simple experimental verification is conducted; the results show that this method has strong robustness in recognizing noisy ultrasonic signals, provides effective solutions, and can be used as a reference for future defect detection.

## 1. Introduction

Concrete is a vital material in construction, with wide applications in practical engineering. Nevertheless, owing to its low tensile strength and influences such as manufacturing processes, environmental temperature variations, prolonged loading, and buried pipes, concrete structures are susceptible to structural damage like cracks, spalling, and voids. These quality defects pose significant risks to the secure and dependable operation of concrete structures. Consequently, regular defect detection is essential to promptly implement effective prevention and repair measures, ensuring the continuous and safe operation of the structures [1].

To avoid impacting the usability and functionality of the detected structure, non-destructive testing methods are often employed to identify internal defects [2,3,4,5,6]. Common non-destructive testing methods include eddy current testing [7], radiographic testing [8], magnetic particle testing [9], ultrasonic testing [5], and so on. Among these methods, ultrasonic testing has emerged as the most commonly used and convenient technique for detecting defects in structures like concrete [10,11]. According to Dolati et al. [12], ultrasonic testing is suitable for detecting defects such as bond defects, interface damage, and corrosion in concrete structures. It has a wide range of applications; the test results are highly reliable due to its strong penetration ability, high sensitivity, low cost, and ease of operation. Array ultrasonic testing (AUT) is a novel industrial non-destructive testing technology developed based on ultrasonic inspection [13,14,15]. By controlling the transmission and reception of multiple ultrasonic transducers in the array, it enables rapid and precise scanning of the target area. In comparison to traditional ultrasonic testing, it can achieve fast and wide-ranging scans without moving the transducer, which provides higher sensitivity and accuracy. AUT is widely utilized in the field of defect detection [16,17,18,19].

However, in engineering practice, the data analysis and defect discrimination of AUT often rely on the subjective judgment of specialized technicians. The automation level of concrete defect detection based on array ultrasonics is relatively low, requiring significant human and material resources and time. To address this challenge, researchers have recently begun exploring structural defect detection combined with deep learning methods. Deep learning methods [20] excel in the handling of large and complex data, offering the potential for automated detection [21]. Many researchers have proposed a variety of effective deep learning models for automated nondestructive testing (NDT) using ultrasonic waves to detect internal defects in structures, but most of these studies are on metal structures [22,23]. These methods and applications offer valuable insights for defect detection in concrete structures [24,25].

The most typical neural network models in deep learning are the backpropagation neural network (BP) [26]; long short-term memory network (LSTM) [27]; the convolutional neural network (CNN) [28], etc. As a classical and widely applied architecture, CNNs [29] are extensively utilized in image recognition, natural language processing, and pattern recognition [30,31,32,33,34]. The localized connectivity, weight-sharing, and pooling operations in CNNs effectively decrease network complexity, reduce the number of training parameters, and demonstrate robustness, fault tolerance, as well as ease of training and optimization [35,36,37,38,39]. These achievements underscore the immense potential of neural networks, particularly CNNs, in recognizing sonic signals. While the anisotropic and heterogeneous properties of concrete pose challenges in defect detection, this approach leverages CNNs to classify and identify the intricate echo signals stemming from defects, thereby facilitating automated localization of these defects within the concrete structure.

Therefore, this study combines AUT with deep learning to extract the underlying features in the learning data, and it aims to achieve automated non-destructive localization of internal hole defects in concrete. The paper proposes a multi-level CNN method capable of distinguishing minute differences between echo signals corresponding to different defects, thereby achieving the objective of defect localization. This work can provide new ideas and solutions for the application of ultrasonic testing technology in the field of defect identification.

## 2. Convolutional Neural Network Algorithm

The CNN was first proposed by Professor LeCun from New York University [28]. Its essence lies in extracting deep-level abstract features from the input data through corresponding convolution operations. In recent years, many researchers have used CNN to detect and classify defects, all of which have shown good recognition performance [40,41,42,43]. Most of them directly extract features from 2D images to train neural network models [44], while only a few studies can perform the defect recognition directly from 1D signals [45]. Due to the time-consuming and computationally intensive nature of image acquisition and processing, this study adopts a feature extraction approach from original time-domain signals. Subsequently, a one-dimensional CNN is employed for identifying and classifying concrete ultrasound signals with internal defects, which achieves automated localization of structural internal defects. For the defect recognition and classification problem of ultrasonic signals, using a CNN algorithm significantly reduces computational complexity and improves computational efficiency.

### 2.1. Basic Principles of Convolutional Neural Networks

**Figure 1:**
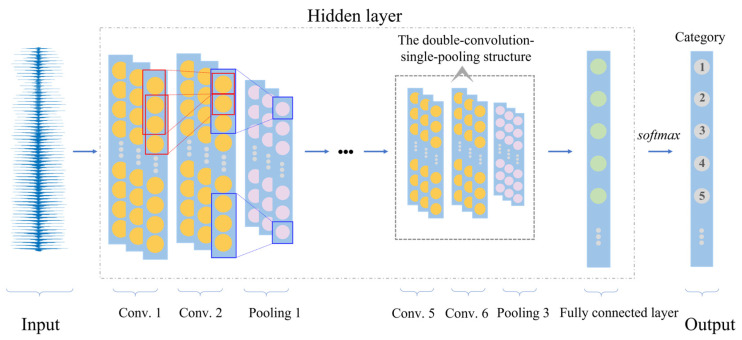
The basic architecture of the CNN.

The CNN employed in this study mainly includes an input layer, hidden layer, and an output layer. The hidden layer consists of several convolutional layers, several pooling layers, and a fully connected layer. A representative structure of CNN is illustrated in Figure 1. The one-dimensional ultrasonic signal is taken as the input of the CNN model, followed by the hidden layer. There are three sets of structures in the hidden layer. Each set consists of two convolutional layers and one max pooling layer. The double-convolution single-pooling structure effectively extracts features from the input data while reducing its dimensionality and complexity through pooling operations, thus enhancing the model’s efficiency and generalization capability. Following this is the fully connected layer, which connects the features from the previous layer and passes the output values to the Softmax activation function. This process outputs the corresponding regional category number of the defect, ultimately resulting in defect localization.

### 2.2. Feature Extraction and Classification

The CNN reduces the number of parameters and computational complexity by performing convolution operations on the feature maps of the previous layer. The convolutional kernel slides over the input feature maps, extracting features at each position using the defined stride. The extracted features are then processed by an activation function and passed to the next layer. Assume there are *U* data groups in the input layer, and each data group has *I* inputs, which can be described by ${\left\{x_{ui}\right\}}_{i=1}^{I}\subseteq {ℜ}^{I}\left(u=1,\ldots ,U\right)$. Define $x_{uj}^{lk}$ (l=1,⋯,L; k=1,⋯,K; u=1,⋯,U; j=1,⋯,J) as the output of the *k*-th convolutional layer of the *l*-th hidden layer, where *L* denotes the number of the double-convolution single-pooling structures and *l* defines the *l*-th structures; *K* is the number of layers corresponding to the current convolutional layer and *k* denotes the *k*-th convolutional layer; and *J* is the number of the nodes, and *j* is the *j*-th node. The expression from the input $x_{ui}^{lk-1}$ to the output of the *k*-th convolutional layer $x_{uj}^{lk}$ is expressed as follows:

(1) $$\begin{array}{l} x_{uj}^{lk}=F\left(\sum_{i=1}^{I}x_{ui}^{lk-1}\times W_{ij}^{lk}+b_{j}^{lk}\right),\\ k=1,\cdots ,K;l=1,\cdots ,L;u=1,\cdots ,U;j=1,\cdots ,J. \end{array}$$

where $x_{ui}^{lk-1}$ represents the input for the *k*-th convolutional layer of the *l*-th hidden layer, $W_{ij}^{lk}$ is the weight matrix, ∗ denotes the convolution operator, and $b_{j}^{lk}$ is the bias term, *F* denotes the activation function in convolutional layers. The *Tanh* function is employed as the activation function.

To further accelerate the computation speed and reduce the number of parameters, max pooling layers are introduced following the convolutional layers. Max pooling reduces the dimensionality of the feature vectors obtained from convolution by selecting the maximum value within each pooling region. The calculation formula for max pooling is expressed as follows:

(2) $${\left\{X_{un}^{lk}\right\}}_{n=1}^{N}=\mathrm{down}\left({\left\{x_{uj}^{lk}\right\}}_{j=1}^{J}\right)$$

Here, ${\left\{x_{uj}^{lk}\right\}}_{j=1}^{J}$ is the input of the pooling layer, which is also the output of the convolutional layer, ${\left\{X_{un}^{lk}\right\}}_{n=1}^{N}$ denotes the output after pooling, and $\mathrm{down}\left(\cdot \right)$ is the pooling function. Commonly used pooling methods include average pooling and max pooling. In this study, the max pooling method was adopted, which can highlight the features of larger and more complex signals without changing the spatial dimension of the data and effectively preserving their location information. The pooling stride length is set to 3, and the expression for max pooling is as follows:

(3) $$X_{un}^{lk}=max\left\{x_{u(3n-2)}^{lk},x_{u(3n-1)}^{lk},x_{u(3n)}^{lk}\right\}$$

In which *n* represents the number of outputs after pooling, which is determined by the size of the input to the pooling layer. $\max\left\{\right\}$ refers to the maximum pooling function.

Assume the output of the last pooling layer as ${\left\{X_{un}^{LK}\right\}}_{n=1}^{N}\subseteq {ℜ}^{N},u=1,\ldots ,U.$ The subsequent step involves introducing a flatten layer to transform the output data, which facilitates classification by the fully connected layer. Let ${\left\{y_{uq}\right\}}_{q=1}^{Q}\subseteq {ℜ}^{Q}\;\left(u=1,\ldots ,U\right)$ be the *Q* outputs of this layer. This process can be represented by the following equation:

(4) $$y_{uq}=\sum_{n=1}^{N}X_{un}^{LK}V_{nq}+c_{q},u=1,\ldots ,U;q=1,\ldots ,Q.$$

where *y_uq_* is the output value of the *q*-th node, *V_nq_* represents the weight connecting the *n*-th neuron node from the previous pooling layer to the *q*-th node of the subsequent fully connected layer, and *c_q_* denotes the corresponding bias term.

In the subsequent step, the Softmax function is employed to align the output vectors with their respective categorical labels in a one-to-one manner, and the cross-entropy loss is then computed. The mathematical expression is as follows:

(5) $$p_{uq}=\frac{e^{y_{uq}}}{\sum_{\zeta =1}^{Q}e^{y_{u\zeta }}},u=1,\ldots ,U;q=1,\ldots ,Q$$

Here, *p_uq_* represents the predicted probability for corresponding category. The Softmax function exponentiates the input, transforming the model’s predictions for each class into a probability distribution within the [0, 1] range, where the probabilities collectively sum to 1. The labeled class with the highest corresponding probability represents the result predicted by the model. Finally, the cross-entropy function is utilized to calculate the prediction error of the model, which is expressed as follows:

(6) $$L=-\frac{1}{U}\sum_{u=1}^{U}\sum_{q=1}^{Q}\left({\bar{P}}_{uq}\log\;p_{uq}\right)$$

where *L* represents the loss function, *p_uq_* denotes the predicted probability for being the *q*-th category, ${\bar{P}}_{uq}$ is the true probability distribution, and *Q* refers to the number of classification labels, *U* is the number of samples. 1/*U* represents the factor utilized in computing the average value, which is essential for deriving the mean of the cross-entropy loss. Here, the cross-entropy function is employed as the loss function for training.

## 3. The Principle of Array Ultrasonic Testing

**Figure 2:**
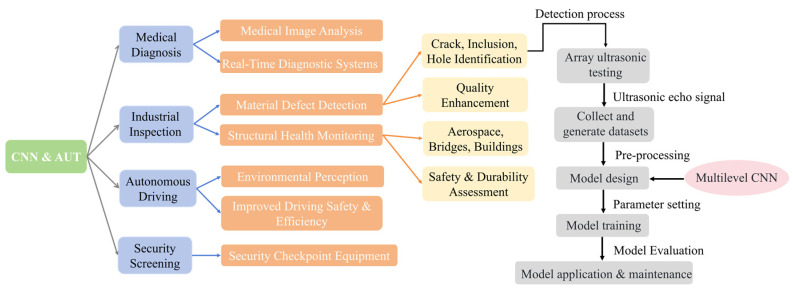
Illustration of CNN–AUT integrated applications and defect detection workflow.

AUT is a novel non-destructive testing technique that combines array probes with traditional ultrasonic testing principles. The array probe is composed of numerous different piezoelectric crystals arranged in a specific sequence, enabling the detection of defects in different positions and orientations within structures. In array ultrasonic testing technology, by arranging the transducers and adjusting parameters, such as the waveform, amplitude, timing, and receiving channels of the transducers, the emitted acoustic waves from each transducer can be received by different transducers. This method provides enhanced functionality and flexibility for ultrasonic testing. Combining it with CNN can fully leverage the advantages of both and demonstrate the potential and advantages of practical applications in various fields. In comparison to other ultrasonic non-destructive testing techniques, especially in the detection of deep-seated structural defects, AUT offers significant advantages such as fast detection speed, wide coverage, and high sensitivity. The application range and detection process in the field of defect identification are shown in Figure 2.

**Figure 3:**
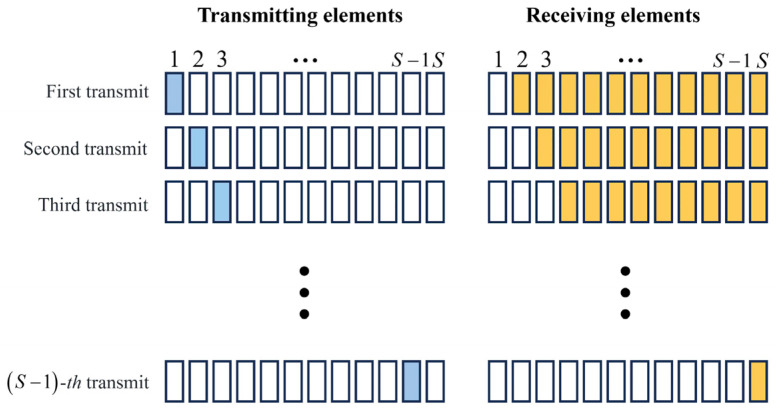
Ultrasonic signal transmission and reception modes.

The data acquisition mode utilized in this study is the half-matrix capture [46], where each element sequentially transmits signals, with non-transmitting transducers responsible for receiving signals. The acquisition principle is illustrated in Figure 3. When element 1 transmits the excitation signal, elements 2 to *S* receive. When element 2 transmits, elements 3 to *S* receive, and so on, until element *S* − 1 transmits and element *S* receives, completing the entire signal transmission and acquisition process. Thus, a complete acquisition only requires capturing signals from *S*(*S* − 1)/2 channels. This is due to the reciprocity theorem in the transmission matrix [47], where equivalent ultrasound paths and characteristics exist in the acquisition channels. Therefore, during data collection, only one from a set of equivalent channels needs to be acquired, significantly reducing the required data collection volume, effectively saving storage space, time, and costs.

## 4. Numerical Studies

This study uses the multi-physics simulation software COMSOL Multiphysics 5.6 to establish a non-destructive testing model for pure, isotropic concrete defects based on AUT. The model specifically simulates the isotropic properties of the concrete medium by assuming homogeneous material properties in all directions, which enables it to simulate ultrasonic propagation and its interaction with internal defects during defect detection. The detection process of anisotropic and heterogenous problems is the same as the isotropic and homogeneous problems. This is because the information, including the defects and materials, is all included in the received signal, and multilevel CNN can effectively identify and distinguish these features in the signal.

Firstly, a two-dimensional cut-off point is added at the top of the model to simulate the transmission and reception of signals by the ultrasonic array probe. After that, pure concrete models with defects in different positions are randomly generated. Echo signal data from the defects at different positions are exported and post-processed in batches, which results in a dataset with a sample size of 8064. This dataset is used for training and classification in subsequent neural network analysis.

### 4.1. Model Establishment

**Figure 4:**
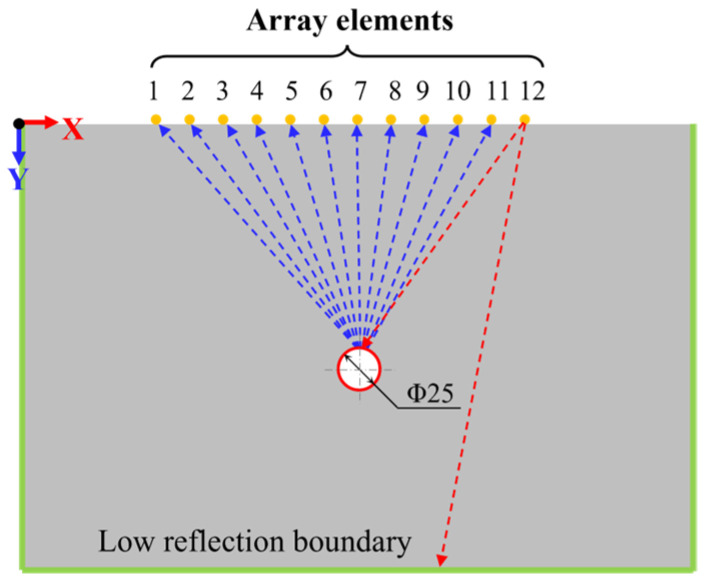
Two-dimensional planar geometric model and signal transmitting/receiving process (The red dotted lines represent the transmitted signal and the blue dotted lines represent the received signals).

Finite element simulation was conducted in COMSOL. A transient study was chosen. Based on the equivalent plane strain condition, the actual three-dimensional spatial problem was simplified to a two-dimensional plane problem. A new two-dimensional model with a solid mechanics physics interface was created for the simulation. As shown in Figure 4, the model is a two-dimensional semi-infinite plane geometry; a domain of 600 mm × 400 mm was utilized for simulations. The material of the model was set as pure concrete with a strength of C30. The model parameters are listed in Table 1. Here, *f_z_* is the frequency of ultrasonic waves, *f_s_* denotes the sampling frequency, *c_S_* refers to the shear wave velocity, *c_L_* is the longitudinal wave velocity, and *ρ* represents the material density, E denotes Young’s modulus, and *μ* is the Poisson ratio. The model contains a circular hole defect with a diameter of 25 mm; the corresponding ultrasonic echo signal was obtained by modifying the position of the defect in the model, so as to obtain an ultrasonic echo dataset with different defect location information.

To simplify the model and improve computational efficiency, 12 equidistant, two-dimensional cut-off points were placed at the top of the model to simulate an ultrasonic phased array. The transducer array was placed at the vertical coordinate *y* = 0, and the horizontal coordinate ranged from *x* = 135 mm to *x* = 465 mm. The spacing between each transducer array was set to 30 mm. Following the data acquisition principle displayed in Figure 3, the ultrasonic pulse signal was transmitted sequentially by each element from right to left (as shown in Figure 4), while the remaining elements on the left side received the echo signals. This process continued until the last element received the echo signal, which completed the comprehensive scan. A total of 66 channels of ultrasonic echo signal data were collected per acquisition. Each channel contained 2048 data points, with a time interval of 2 × 10^−5^ s between each data point acquisition. Consequently, the total number of points in a set of data across all channels amounted to 66 multiplied by 2048, which equals 135,168.

**Figure 5:**
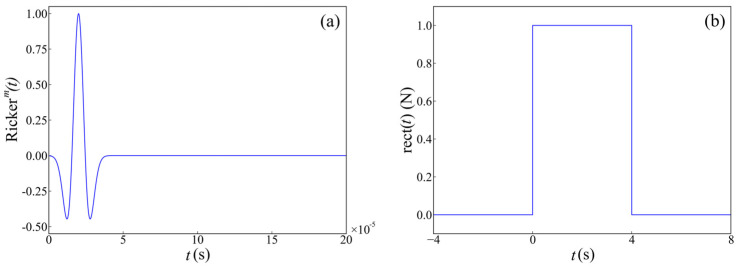
Ultrasonic excitation pulse signal: (**a**) Ricker wavelet; and (**b**) rectangular wave.

The ultrasonic excitation pulse signal was applied in the form of a point load, with the application direction parallel to the *x*-axis and the upper surface of the model. This arrangement ensured that the vibration direction of the particle was perpendicular to the direction of wave propagation, which simulated vertically polarized shear waves. In this study, the Ricker wavelet [48] was used to simulate the ultrasonic excitation load. The time-domain waveform is shown in Figure 5a, and its expression is represented as follows:

(7) $$\mathrm{rect}\left(t\right)=\left\{\begin{array}{cc} 1, & t,\left[0,4\right)\\ 0, & \mathrm{others} \end{array}\right.$$

(8) $$\mathrm{Ricker}(t)=\left\{1-2{\left[\pi f_{z}\left(t-t_{0}\right)\right]}^{2}\right\}\exp\left\{-{\left[\pi f_{z}\left(t-t_{0}\right)\right]}^{2}\right\}\mathrm{rect}\left(t\right)$$

where *f_z_* is the center frequency with a magnitude of 50 kHz, *t*_0_ denotes the time offset with a magnitude of 20 microseconds, rect(*t*) corresponds to a rectangular wave, as depicted in Figure 5b.

**Figure 6:**
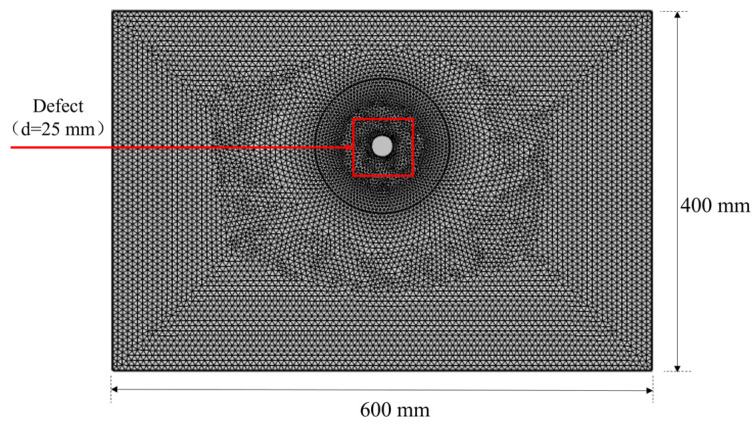
Finite element mesh.

This study employs time-domain numerical simulation to analyze the propagation characteristics of ultrasonic waves in concrete. In order to mitigate the interference caused by reflected waves and acoustic mode conversion on the propagating sound field, low-reflection boundary conditions were applied to the ends and bottom of the computational region, while the remaining boundaries were set as free boundaries. A free triangular mesh was utilized for grid partitioning in the numerical model. The specific grid partitioning is illustrated in Figure 6. The maximum element size was set to one-tenth of the wavelength, which corresponds to 4.56 mm. To obtain a larger number of data samples, improve computational efficiency, and ensure accuracy, a circular region was defined around the defect. Coarser grids were employed outside this region, while finer grids were employed within the region. This configuration aims to ensure the effectiveness of the model simulation while reducing the computation time for each model and meets the practical requirements of obtaining a large number of data samples in this work.

### 4.2. Multilevel Classification Method

Given the complexity and diversity of the data, particularly when considering limited computing capabilities, there is a constant need for efficient and precise defect identification methods. Therefore, this work introduced a multilevel CNN method based on length scales. The main idea of this method is to improve the recognition accuracy and efficiency of neural networks by identifying and locating defects within the model’s local regions at each level. The number of levels, the number of regions per level, and the size of each region can be determined based on the model’s size and the neural network’s effectiveness in defect recognition. The size of each region is selected as the same, and the regions within each level should not overlap and collectively cover the entire detection area. Dividing the model detection area into *γ* levels, if the defect recognition accuracy meets the target requirements and adding an additional classification level does not significantly improve the precision of recognition, while reducing the classification level has a significant impact on recognition precision, then the current classification level is considered the most suitable. The size of each region within the level can be determined according to practical considerations, which ensures both computational efficiency and accuracy. The general guideline followed is that, in order to reduce the workload and improve recognition efficiency, the regions in the earlier levels should be larger, while the size of the classification region in the final level should be determined based on the acceptable detection accuracy error, which is preferably as small as possible.

**Figure 7:**
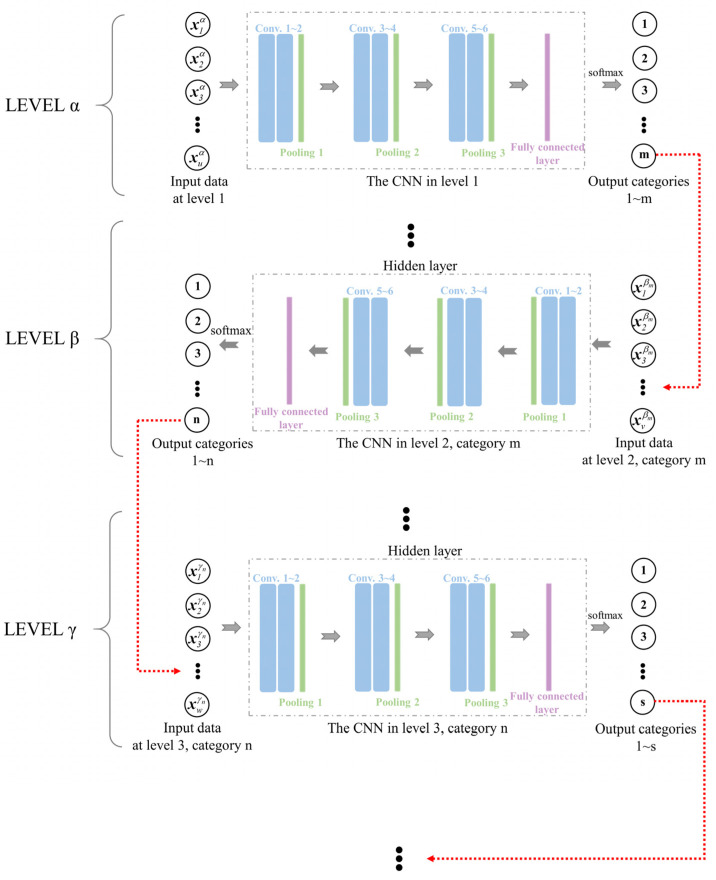
The structure of the multilevel CNN (*m*, *n*, *s*, … represent the number of classification categories for the corresponding levels 1, 2, 3 …, respectively. *u*, *v*, *w* represent the quantity of input data. *α*, *β*, *γ* represent the number of levels. $x_{u}^{\alpha }$ is the *u*-th input of level *α*. $x_{v}^{\beta m}$ is the *v*-th input of level *β*, belonging to the *m*-th region of level *α*. $x_{w}^{\gamma n}$ is the *w*-th input of level * γ*, belonging to the *n*-th region of level *β*).

**Figure 8:**
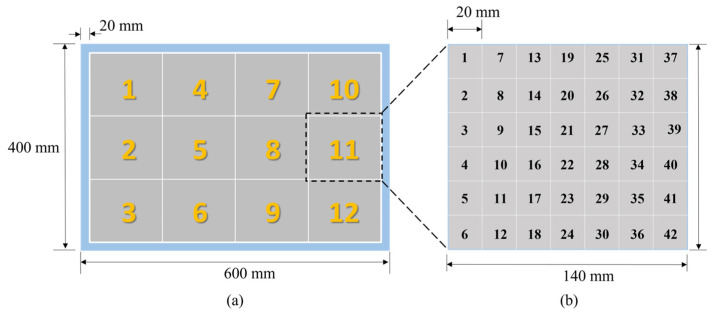
Schematic diagram of model division and classification: (**a**) the first size level; and (**b**) the second size level.

The structure of the multilevel CNN is illustrated in Figure 7. In this study, two size levels were established, with consideration given to both the dimensions of the detection model and the required recognition accuracy. To mitigate boundary effects, a 20 mm-wide boundary region was preserved around the entire model. The internal area, with dimensions of 560 mm × 360 mm, was designated for accommodating randomly generated circular defect patches. The first size level divided this area into 12 blocks, with each block having a classification area size of 140 mm × 120 mm. These 12 blocks were further subdivided into 42 smaller squares, each measuring 20 mm × 20 mm. The specific division is displayed in Figure 8. Then CNN models were established in the first level and in the 12 regions of the second level. Each CNN model consisted of three double-convolution single-pooling structures.

**Figure 9:**
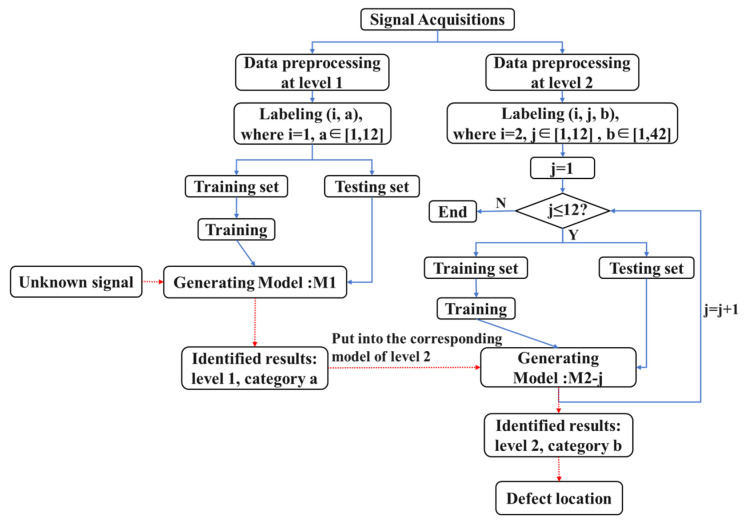
Flowchart of the multilevel classification method.

Subsequently, multiple sets of signals were input into this network structure for model learning, and the models were trained in the two size levels through iterative parameter updates to minimize the loss function. This process ultimately yielded a defect detection model with the desired recognition accuracy. The flowchart for the proposed method is exhibited in Figure 9. When identifying and locating defects, the first step is to determine their classification position within the first level. Once confirmed, the corresponding block is further subdivided into smaller-sized model blocks, referred to as the second level. Within this size level, the position is refined even further. Finally, combining the results of these two recognition stages enables the precise determination of the defect’s specific location. By applying the multilevel method into the recognition of defect ultrasonic signals within a CNN, computational resources can be effectively utilized, leading to reduced computation time and enhanced classification accuracy.

### 4.3. Data Acquisition

**Figure 10:**
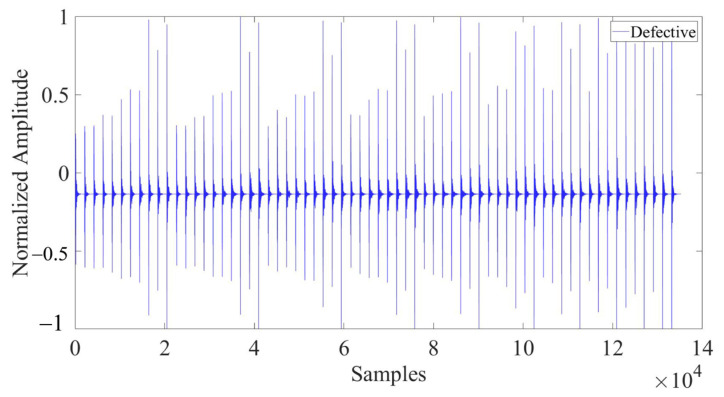
Numerically simulated ultrasonic echo signal with defect center at X = 300 mm, Y = 200 mm.

The COMSOL with MATLAB was employed for collaborative computing in this study. To automate the process, relevant code programs were developed to modify the defect positions in batches and subsequently export the corresponding echo signal data. A total of 8064 sets of ultrasonic echo signal data with different defect positions were collected. Subsequently, the 66-channel data from each set were merged into one-dimensional data, 1 × 135,168 in size. As shown in Figure 10, the ultrasonic echo signal in the figure was collected from a finite element model with the defect center located at X = 300 mm, Y = 200 mm.

**Figure 11:**
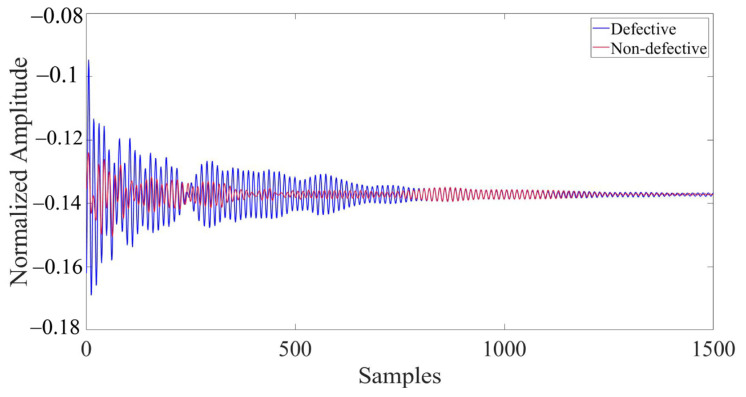
Comparison of partial waveforms with and without defects.

**Figure 12:**
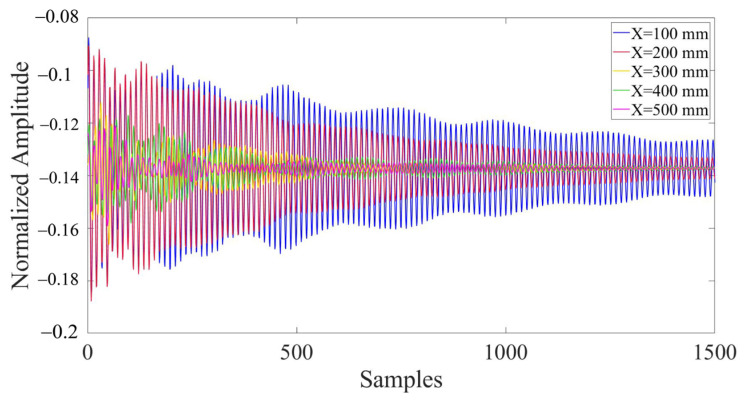
Comparison of partial waveforms with different defect horizontal coordinates (Y = 200 mm).

**Figure 13:**
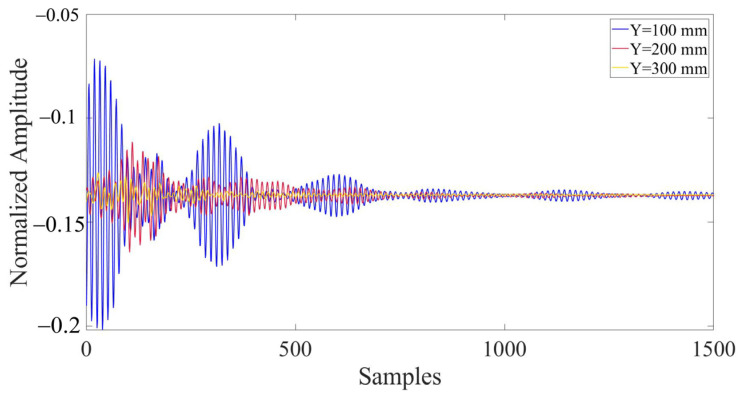
Comparison of partial waveforms with different defect vertical coordinates (X = 300 mm).

In the model, the presence of defects and their varying locations result in significant differences in the collected ultrasonic waveforms. Due to the vast amount of data contained in the complete waveform, it is difficult to directly display and highlight the differences in waveforms under different defect conditions. Therefore, 1500 representative data nodes were selected and enlarged from the entire waveform, which comprised 135,168 data nodes, for the purpose of comparison. Figure 11 depicts the comparison of partial waveforms when there are defects and no defects within the concrete model. Figure 12 represents the partial waveforms when the horizontal coordinates of the defects differ, and Figure 13 illustrates the differences in partial waveforms when the vertical coordinates of the defects vary. In a set of ultrasonic echoes like these, there are hidden relevant information elements containing defect features. CNNs can effectively extract these elements, thereby capturing the essential differences in the echo signals collected from different defect locations and achieving the goal of defect localization.

Before training the CNN model, each group of data needs to be labeled with corresponding category numbers, based on the model partition principle in Figure 8. In the first size level, 12 regions are sequentially numbered from top to bottom and left to right: the first column is labeled 1 to 3, the second column is labeled 4 to 6, and so on, with the last column labeled 10 to 12. Therefore, the 8064 groups of data can be correspondingly matched with the numbers based on the defect location regions, with each category containing 672 groups of data. After completing this step and labeling the data accordingly, the preparation of the first-level dataset was completed. In the second size level, the original 8064 groups of data were precisely divided into 12 subsets based on the region division in the first level. Taking the first region of the first level as an example, this area contained 672 sets of ultrasonic echo signal data, all of which were obtained from concrete models with defects located in area 1. According to the previous division, this region contains 42 smaller blocks, with each block collecting 16 sets of data. The 42 regions were then sequentially numbered from top to bottom and left to right: the first column was numbered 1 to 6, the second column is numbered 7 to 12, and so on, with the last column numbered 37 to 42. After numbering, each group of data was labeled with the corresponding region number representing the defect position in the concrete. By following these steps, the dataset preparation for the first region in the second size level was completed. Using the same processing flow, the preparation work for datasets of the remaining regions was conducted sequentially.

### 4.4. Training Process and Result Analysis

The hyperparameters for model training are shown in the Table 2. The initial learning rate was set to 0.01, the optimizer was Adam, the loss function was chosen as cross-entropy loss, the batch size for each training iteration was 32, and the number of epochs was set to 15.

**Figure 14:**
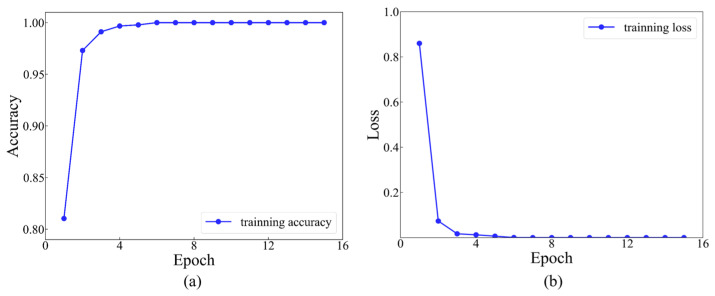
Training accuracy and loss function curves at level 1: (**a**) training accuracy at level 1; (**b**) training loss at level 1.

In the first size level, a dataset comprising 8064 sets of data was labeled, with each set assigned a label ranging from 1 to 12. Each category contained 672 sets of data. Afterwards, the dataset was randomly divided into a training set, a validation set and a testing set, with a ratio of 8:1:1. The model stopped training when the value of the training loss function was under 10^−5^. The iterative training process was recorded for the initial 15 epochs. The accuracy and loss functions during the training process of 15 epochs are indicated in Figure 14.

**Figure 15:**
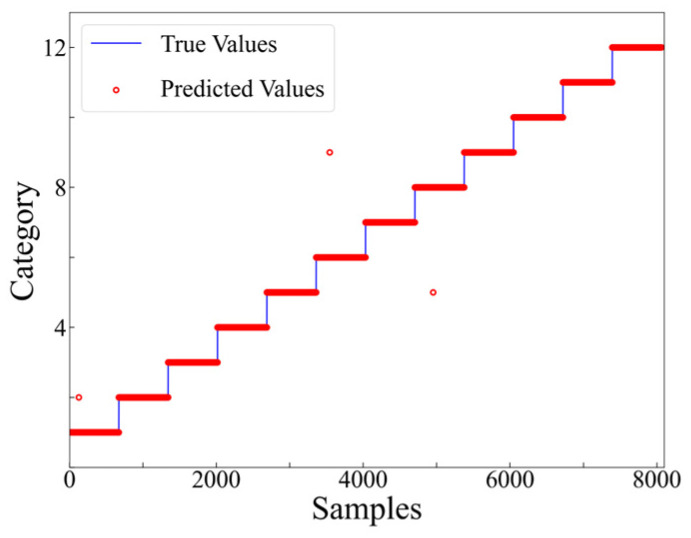
Prediction results at level 1.

As presented in Figure 14, it is evident that commencing from the 6th training epoch, the accuracy has achieved 100%, while the value of the training loss function has diminished to below 0.01. This indicates that the results are convergent and have successfully learned the features within the dataset. The final accuracy of the testing set, which consists of 806 samples, is 99.63%, with only 3 misclassified samples (as shown in Figure 15).

**Figure 16:**
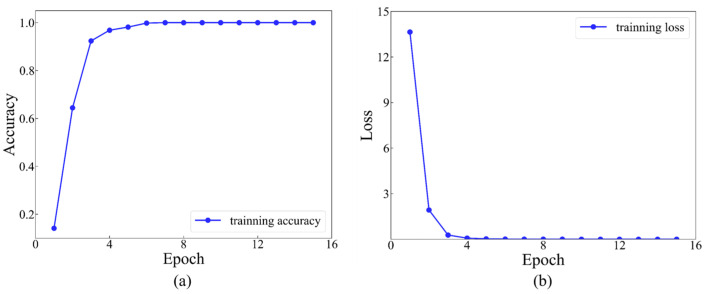
Training accuracy and loss function curves at level 2, category 1: (**a**) training accuracy at level 2, category 1; and (**b**) training loss at level 2, category 1.

In the second size level, the initial 12 blocks were further subdivided into 42 smaller blocks. Each of these blocks contained 672 sets of data, labeled sequentially from 1 to 42, with each category consisting of 16 sets. Similar to the first size level, the dataset was divided into a training set, a validation set, and a testing set; the ratio was 8:1:1. When the value of the training loss function was less than 10^−3^, the model stopped training. The iterative training process, spanning 15 epochs, was documented. Consequently, trained CNN models were acquired for each of the 12 larger blocks, accompanied by their respective accuracy and loss function metrics throughout the training phase. Here, the analysis focused on the accuracy and loss function of the training process within the 1st region of the first size level, as exhibited in Figure 16.

**Figure 17:**
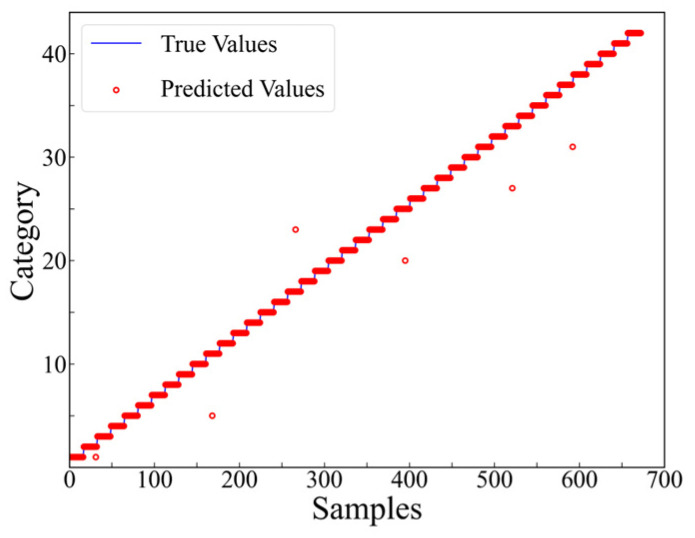
Prediction results at level 2, category 1.

**Figure 18:**
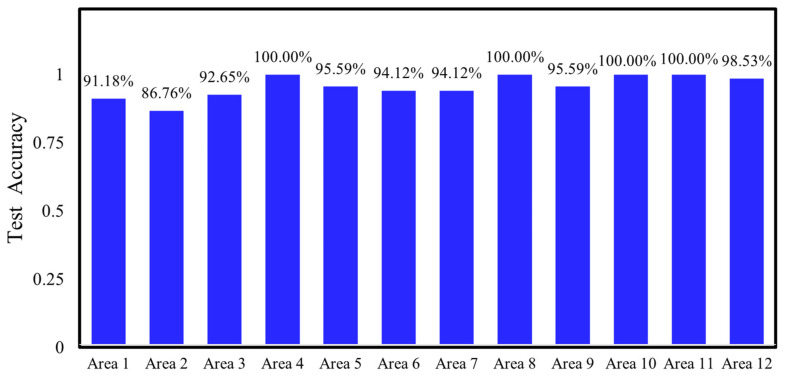
Test accuracy of 12 regions.

**Figure 19:**
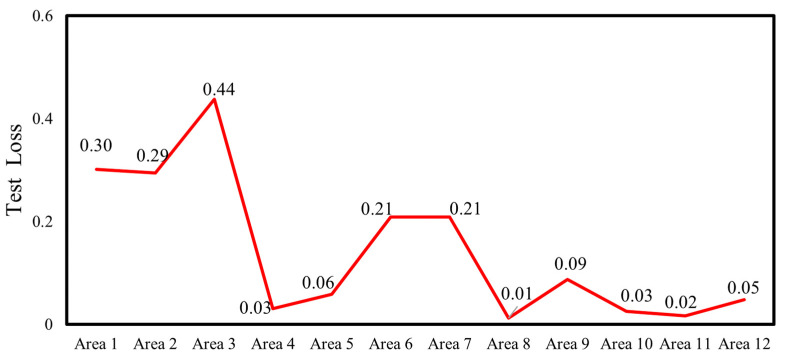
Test loss of 12 regions.

After the 6th training epoch, the accuracy on the training set reached 100%, which indicated that the model had successfully converged. The well-trained model was applied to predict the dataset, and the prediction results for level 2, category 1 are presented in Figure 17. The number of mispredictions amounts to 6. The same trend can be observed in other regions. The accuracy and loss functions on the testing set for the 12 regions, aggregated, and analyzed, are demonstrated in Figure 18 and Figure 19.

To validate the effectiveness of the proposed multilevel classification method in this study, an identical dataset was used to compare its model training outcomes with the traditional CNN method, evaluating both the defect localization accuracy and computational efficiency.

First, the detection accuracies of the multilevel CNN method and the traditional CNN method were analyzed and compared. Both methods utilize the same basic network architecture and hyperparameter settings, with the dataset consisting of the ultrasonic echo signals acquired from the previously mentioned numerical simulation models. A comparison of the accuracies achieved by the two methods is presented in Table 3. It can be observed that for the same dataset with a sample size of 8064, the traditional CNN method divides the data into a training set, a validation set, and a testing set in an 8:1:1 ratio. The testing set consists of 806 samples, and the number of misclassified predictions is 118, which results in an accuracy of 85.38%. In contrast, with the multilevel classification CNN method, 67 sets of data are randomly selected from the second-level 12 regions of the dataset as the testing set, which results in a testing set size of 804. At the first level, there are three misclassified predictions. For the correctly classified data at the first level, they are then passed to the corresponding regions in the second level for further prediction. The number of misclassified predictions for each region in the second level are as follows: 6, 9, 5, 0, 3, 4, 4, 0, 3, 0, 0, 1. Overall, the method has a total of 38 misclassified predictions, with an accuracy of 95.27%. By adopting the multilevel CNN method, the accuracy improves from 85.38% to 95.27%, with an improvement of 9.89% compared to the neural network without the multilevel classification method.

Second, the time-consuming performance of the two methods was comparatively analyzed by recording the CPU runtime during model training and prediction for both methods, with the process repeated five times and the results averaged. As depicted in Table 4, the traditional CNN approach necessitates training and recognizing data across 504 categories, consuming approximately 14,257 s for training. For an unknown ultrasonic signal, predicting the corresponding defect location requires roughly 7.09 s, resulting in a sluggish computation process that heavily consumes computational resources, rendering the model cumbersome and intricate. In contrast, the multilevel CNN method exhibits a training time of 11,236 s at the first level. Within the second level, each sub-region’s model, corresponding to a specific category, trains in merely 933 s. The prediction of a single data point consumes just 3.15 s at the first level and an additional 3.11 s at the second level, translating to an 11.71% improvement in prediction speed. Therefore, when confronted with vast datasets, the multilevel CNN demonstrates the capability to swiftly and accurately classify and identify detection targets, offering more stable and superior performance in defect localization tasks.

## 5. Experimental Case Study: Localization of Hole Defects in Concrete

To further validate the effectiveness of the proposed method for the detection of internal defects in concrete structures, experimental studies were conducted. Internal defective concrete was employed as the target object, and ultrasonic echo signals from various defect locations were collected to form the training dataset. The dataset was annotated with corresponding position labels. Subsequently, these labeled data were utilized for training and recognition in the CNN established in this study.

**Figure 20:**
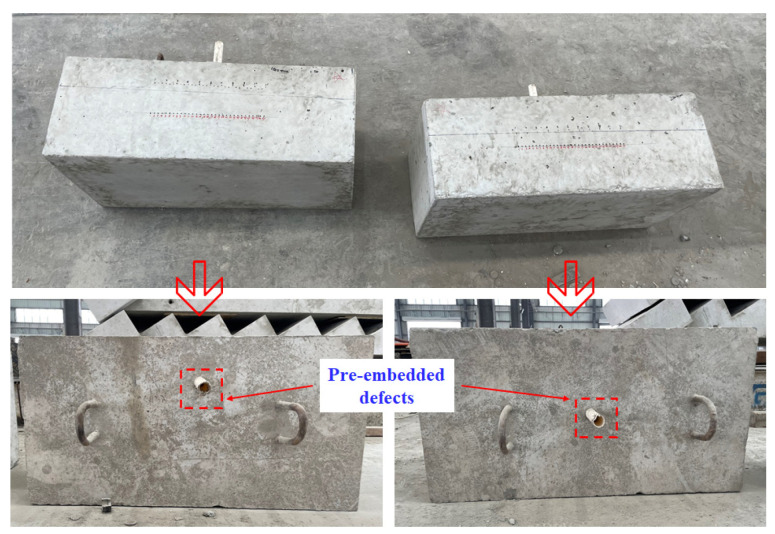
Concrete specimens with defects at varying depths.

Considering the data requirements and engineering implementation conditions, this experiment took pure concrete specimens with a cross-sectional size of 800 mm × 400 mm as the research object. The strength of the concrete specimens produced was C30. The internal circular hole defects were made by embedding PVC pipes, and the diameters of the hole defects were set to 25 mm, as shown in Figure 20. All specimens had the same raw material ratio and manufacturing process and were cured under standard curing conditions for 28 days.

**Figure 21:**
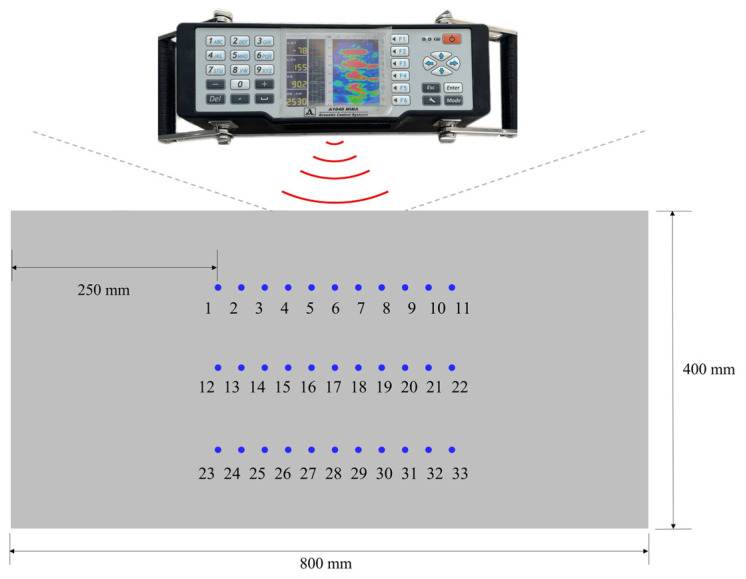
The location of defects for ultrasonic signal collection.

This experiment selected different horizontal positions of hole defects located at three representative depths for ultrasonic echo data collection. The three representative defect depths were 100 mm, 200 mm, and 300 mm. At each depth, the hole defects were set at 11 different horizontal positions. At each depth, the interval between two adjacent positions was 30 mm. After that, the echo data were collected when the defects were located at these different positions. As shown in Figure 21, a total of 495 sets of ultrasonic echo signals were collected from 33 different locations of the hole defects, with 15 sets of data collected at each location.

**Figure 22:**
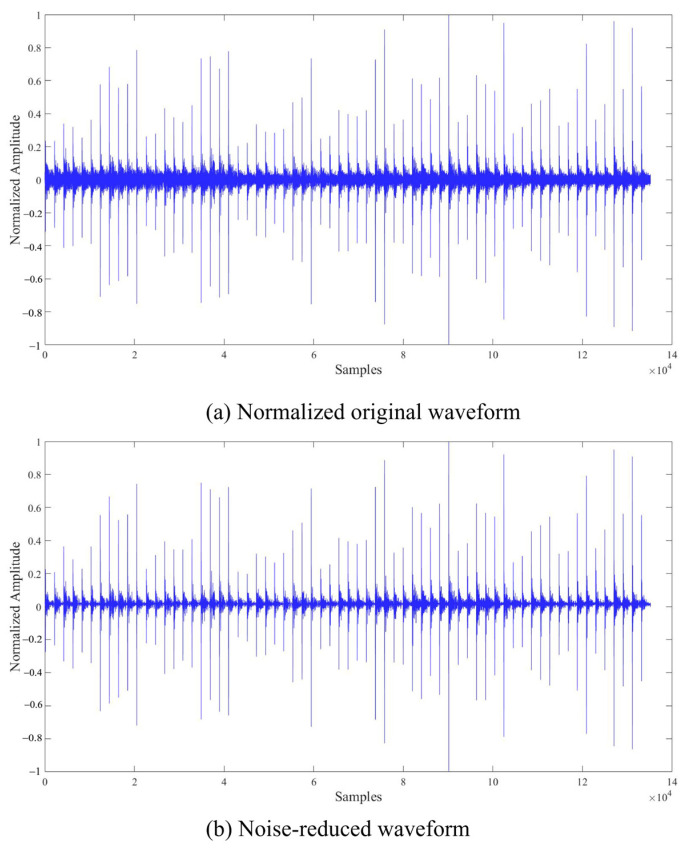
The comparison of waveforms before and after noise reduction.

Due to the significant noise interference in the experimental waveforms, wavelet denoising was employed to smooth and reduce noise in each set of data, aiming to enhance the clarity and accuracy of the signals. The detailed comparison of the experimental waveforms before and after processing is provided in Figure 22.

Based on the defect location, a multilevel classification approach was utilized to distinguish the experiment dataset of defect identification into two levels, as follows: the first level included three categories of defects with depths of 100 mm, 200 mm, and 300 mm. A total of 495 datasets were labeled D1, D2, and D3, based on different depths from top to bottom across 33 positions. At the second size level, the defect location categories were further refined for each of these three depth types. Specifically, for each depth, there were 165 sets of defect echo signals corresponding to 11 different horizontal positions from left to right, labeled as H1 to H11, respectively. Finally, the labeled dataset was randomly shuffled and utilized to train the proposed multilevel CNN, aiming to obtain well-trained models for both the first and second classification levels. Due to the relatively limited availability of experimental datasets, in order to ensure that the model could learn sufficient features during the training process and to increase the data volume of the testing set as much as possible for a more comprehensive evaluation of the model’s performance in practical applications, the division ratio between the training set and the testing set in the experimental dataset was adjusted. Specifically, a 7:1:2 ratio was adopted for training, with 70% of the data used as the training set, 10% of the data as the validation set, and 20% as the testing set. The model stopped training when the value of the training loss function is below 1 × 10^−7^. The training process for the first 15 cycles was recorded for each level.

**Figure 23:**
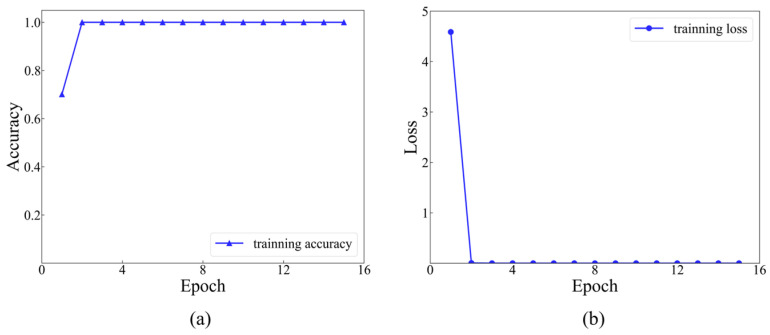
Experimental training accuracy and loss function curves at level 1: (**a**) experimental training accuracy at level 1; and (**b**) experimental training loss at level 1.

**Figure 24:**
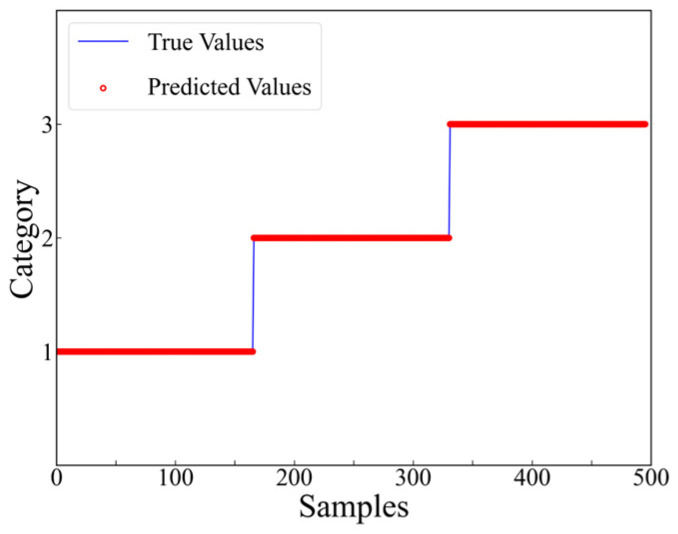
Experimental prediction results at level 1.

**Figure 25:**
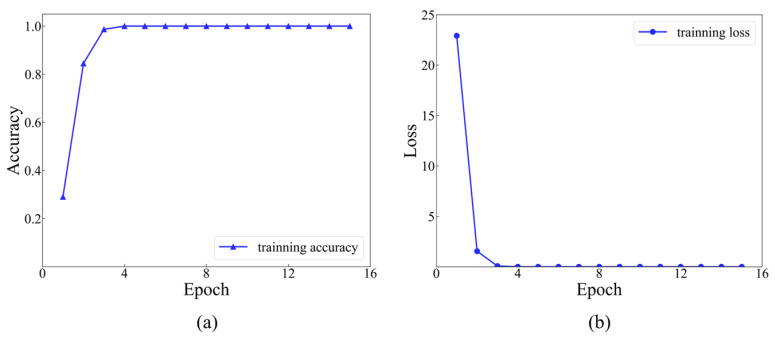
Experimental training accuracy and loss function curves at level 2, depth 1: (**a**) experimental training accuracy at level 2, depth 1; and (**b**) experimental training loss at level 2, depth 1.

**Figure 26:**
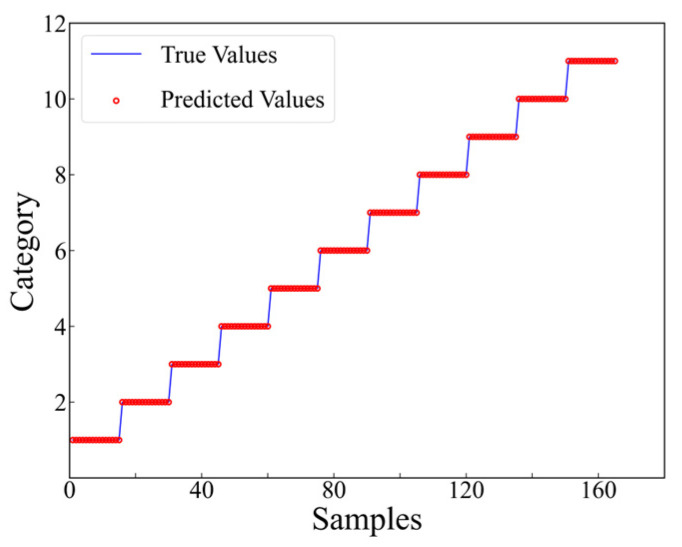
Experimental prediction results at level 2, depth 1.

The training accuracy and loss function curves at the first level are indicated in Figure 23. The results demonstrate that the model converged after the second iteration. After 15 epochs of training, the model achieved an accuracy of 100.00% and the loss function value was 1.9738 × 10^−4^. Afterwards, the well-trained model was applied to predict the dataset, and the experimental prediction results at level 1 are displayed in Figure 24. Since the training process for the three depths in the second level is similar, the primary focus was on analyzing the training accuracy and loss function for level 2, depth 1, as shown in Figure 25. It can be observed that the model tends to converge after approximately five iterations. It ultimately achieved an accuracy of 100.00% with a loss function value of 3.9408 × 10^−6^. When the well-trained model was applied to predict the dataset, the experimental prediction results at level 2, depth 1 were obtained, as shown in Figure 26.

Based on the aforementioned results, the proposed multi-level classification method demonstrates excellent recognition performance in this experimental case. In both the first and second levels, the prediction results for the testing set are consistent with the actual values. Even when faced with experimental waveform signals with strong noise, the multi-level classification CNN method is still capable of accurately identifying and classifying waveform signals from different defect locations, achieving the goal of defect localization. This indicates the strong robustness of this method to noise. It also proves the feasibility of this method for practical detection.

## 6. Conclusions and Future Work

In this work, the widely used AUT was combined with a multilevel CNN for concrete defect localization. This method effectively distinguishes ultrasonic echo signals from different holes within concrete, enabling the automated localization of internal defects in concrete. The advantage of this method is that there is no need to manually process the signal and identify the defect. By inputting the original ultrasonic echo signal into the multilevel CNN model established in this study, the hole defects can be tracked layer by layer. In the first step, a finite element model of pure concrete was established, and 8064 sets of ultrasonic echo signals of different defect locations were collected by AUT. Second, a multilevel CNN classification method based on the above data sets was proposed, which divided the detection region based on the length dimension, and each region after partitioning corresponded to a classification category. This method can refine the location of defects step by step, thus improving the accuracy of waveform classification and defect location in different regions. By comparison, the performance of the multilevel CNN method proposed in this paper was better than the traditional CNN; the accuracy rate was increased from 85.38% to 95.27%, and the computational efficiency was also improved. Finally, this method underwent a simple verification in a pure concrete hole defect localization experiment. The experimental results show that the proposed method is robust, accurately classifying the ultrasonic echo signal with actual noise, thus locating the hole defects in the concrete. The results indicate that the method demonstrates reliability and stability in practical applications.

In future research, the following directions will be pursued to expand upon our current work. First, the optimization of model parameters and architecture will continue, with considerations given to integrating the model with other neural networks to achieve enhanced recognition performance. Second, a more comprehensive dataset will be acquired from field applications to further investigate the identification and detection of various internal defects within complex structures, such as reinforced concrete and steel-plate concrete, thereby elevating the method’s practical engineering value. Third, a real-time detection model will be established that is capable of automatically collecting and processing information in practical applications and adjusting the model in real time based on this information. This iterative optimization process will yield a continuously refined detection model, ultimately enabling fully automated localization of internal defects within concrete structures.

## Figures and Tables

**Table 1:** Input parameters of FEM.

*f_z_* (kHz)	*f_s_* (kHz)	*c_S_* (m/s)	*c_L_* (m/s)	*ρ* (kg/m^3^)	E	*μ*
50	1000	2281.7	3526.5	2300	2.73 × 10^10^	0.14

**Table 2:** Hyperparameter settings for CNN.

Description	Values
Learning Rate	0.01
Activation function	Tanh
Batch size	32
Epoch	15
Optimizer	Adam
Loss function	Categorical cross-entropy

**Table 3:** Comparison between two methods of the localization results.

			Total Dataset	Testing Set	Accuracy	Loss	Number of Prediction Errors	Total Number of Prediction Errors
The multilevel classification CNN method	The first level		8064	804	99.63	0.0059	3	38
	The second level	Area 1	672	67	91.18	0.2467	6	
		Area 2	672	67	86.76	0.3060	9	
		Area 3	672	67	92.65	0.4643	5	
		Area 4	672	67	100	0.0170	0	
		Area 5	672	67	95.59	0.1392	3	
		Area 6	672	67	94.12	0.2271	4	
		Area 7	672	67	94.12	0.1508	4	
		Area 8	672	67	100	0.0013	0	
		Area 9	672	67	95.59	0.0810	3	
		Area 10	672	67	100	0.0124	0	
		Area 11	672	67	100	0.0109	0	
		Area 12	672	67	98.53	0.0315	1	
The traditional CNN method			8064	806	85.38	3.7743	118	118

**Table 4:** Comparison of the time consumption between the two methods.

		Training Time (s)	Inference Time (s)
The multilevel classification CNN method	The first level	11,236.13	3.15
	The second level (per category)	933.28	3.11
The traditional CNN method		14,257.31	7.09

## Data Availability

Data are contained within the article.
